# Direct Detection of KPC Peak from Positive Blood Cultures Using MALDI-TOF MS: Are We There Yet?

**DOI:** 10.3390/antibiotics12030601

**Published:** 2023-03-17

**Authors:** Natália Kehl Moreira, Camila Mörschbächer Wilhelm, Aymê Duarte Echevarria, Fabiana Caroline Zempulski Volpato, Priscila Lamb Wink, Afonso Luís Barth, Juliana Caierão

**Affiliations:** 1 Programa de Pós-Graduação em Ciências Farmacêuticas, Faculdade de Farmácia, Universidade Federal do Rio Grande do Sul, Porto Alegre 90610000, Rio Grande do Sul, Brazil; 2 Laboratório de Pesquisa em Bacteriologia Clínica, Universidade Federal do Rio Grande do Sul, Porto Alegre 90610000, Rio Grande do Sul, Brazil; 3 Laboratório de Pesquisa em Resistência Bacteriana, Hospital de Clínicas de Porto Alegre, Porto Alegre 90035007, Rio Grande do Sul, Brazil; 4 Graduação em Biomedicina, Universidade Federal de Ciências da Saúde de Porto Alegre, Porto Alegre 90050170, Rio Grande do Sul, Brazil

**Keywords:** blood culture, KPC, MALDI-TOF MS

## Abstract

Detecting carbapenemase-associated carbapenem resistance is a subject of major clinical and epidemiological concern as it influences therapeutic choice. Matrix-assisted laser desorption ionization time-of-flight mass spectrometry (MALDI-TOF MS) has been proposed as a means to assess bacterial resistance mechanisms. We aimed to detect the KPC enzyme directly from positive blood cultures using MALDI-TOF MS. To do so, 102 clinical *Enterobacteria* were evaluated, including 59 *bla*_KPC_ positives. Proteins were extracted using formic acid, isopropyl alcohol, and water (17:33:50) and spotted onto a steel target plate using the double-layer sinapinic acid technique. Two parameters were considered: (i) the visual detection of a clear peak with the expected KPC *m*/*z* and (ii) the evaluation of the relative intensity of the ions in the peak. A peak was observed in 56/59 *bla*_KPC_-positive isolates (94.9% sensitivity), with no false-positive results (100% specificity). When considering intensity, with a cut-off ≥120 (a.u.), sensitivity was 94.9% and specificity was 95.3%. We proposed a “buffer” zone, with intermediate values of intensity (115 to 125) reaching 100% sensitivity and specificity. The detection of KPC peaks directly from positive blood cultures using MALDI-TOF MS is feasible and rapid, which may improve appropriate patient therapy and antimicrobial stewardship.

## 1. Introduction

Antimicrobial resistance is considered by the World Health Organization (WHO) as one of the top ten global public health threats facing humanity [1]. Carbapenem-resistant *Enterobacterales* (CRE) are one of the main clinical challenges, as they are associated with significant morbidity and mortality, making them priority pathogens for research and the development of new antimicrobials [2,3,4]. When causing bloodstream infections, these bacteria are associated with a 75% increase in in-hospital mortality [5]. Surely, early appropriate antibiotic use is necessary to improve the prognosis of bloodstream infections, as the median survival rate decreases by 7.6% per hour of delay in implementing proper treatment for septic patients [6,7]. Thus, in the context of microbiology laboratories, rapid bacterial identification and detection of antimicrobial resistance from blood cultures play a significant role in guiding the most appropriate antibiotic therapy, aiming to improve patient outcome [8] and highlight antimicrobial stewardship [9,10].

In some cases, detecting the mechanism of resistance is necessary to establish an optimized treatment [11]. When it comes to CRE, carbapenemase production is the main mechanism, and *Klebsiella pneumoniae* carbapenemase (KPC) is the most widespread enzyme in many regions [2,12]. Several approaches have been described for the detection of carbapenemases [13,14,15,16,17]. Traditional microbiological methods such as the combined disc test using carbapenem and carbapenemase inhibitors, such as phenylboronic acid (PBA) and ethylenediaminetetraacetic acid (EDTA), remain convenient as they are easy and cheap to perform and allow to discriminate enzyme type; however, it is not fast enough, as it takes overnight incubation in order to provide results [16]. The carbapenem inactivation method (CIM) and its derivatives, mCIM and eCIM, also require a long turnaround time (20–22 h). On the other hand, although rapid (up to 4 h), colorimetric tests (Carba-NP and Blue Carba) do not discriminate by enzyme type and can also be subjective as they rely on visual observation to determine positivity for hydrolysis [17,18,19]. Polymerase chain reaction (PCR) is a fast and reliable way to detect and discriminate enzymes, but it is unable to detect novel carbapenemase genes. Besides, the correlation of the PCR result with the phenotypic expression of the enzyme may be an issue, as high Ct (threshold cycle) values can miss low-level expression of carbapenemase genes [13,20,21]. Moreover, a considerable portion of clinical laboratories do not include PCR as part of their routine. Another possibility for rapid carbapenemase detection are the immunochromatographic tests, which may detect specific enzymes directly from blood cultures in around 30 min or less [22,23]. However, their costs may be an issue for many laboratories [24].

Matrix-assisted laser desorption ionization time-of-flight mass spectrometry (MALDI-TOF MS) has proven to be an important tool in clinical microbiology laboratories, providing accurate and very rapid bacterial identification. Recently, MALDI-TOF MS has been proposed to assess bacterial resistance mechanisms [25,26,27,28] with a low cost per analysis [17]. Current studies have demonstrated the possibility of predicting carbapenemase production based on the detection of the hydrolyzed carbapenem molecule using MALDI-TOF MS. Although reliable, this detection is laborious, as it demands many extraction steps [14,28,29,30]. Besides, unspecific hydrolysis of the antibiotic may be a concern, compromising specificity; on the other hand, enzymes with reduced hydrolytic activity, such as some oxa-type carbapenemases, could generate false-negative results, reducing sensitivity [31,32].

In order to detect some specific peak that could be related to KPC production, a few authors have proposed that the presence of the KPC enzyme can be predicted at 11,109 Da, regarding the protein p019. However, this method relies on genetic context, as the *p019* gene, present in *bla*_KPC-2-_producing *K. pneumoniae*, belongs to the specific ST258 clone. Therefore, negative results should be complemented by confirmatory tests [33,34,35].

Considering that, the direct detection of β-lactamases by MALDI-TOF MS seems to be a more reliable option. Indeed, recently, methodologies have been developed and evaluated for direct detection of specific β-lactamases, including KPC, using MALDI-TOF MS with promising results [36,37,38,39]. However, a significant number of studies are available on this issue, especially considering the direct detection from positive blood bottles, excluding the culture step. Indeed, to the best of our knowledge, there is a single study that used MALDI-TOF MS to detect KPC from blood cultures [37], which found 100% sensitivity and specificity. However, it evaluated a reduced number of blood culture bottles (39 samples, with only 16 KPC-producing bacteria). To strengthen the usefulness of this methodology, we evaluated an optimized MALDI-TOF MS protocol aiming to detect the KPC enzyme directly from positive blood cultures.

## 2. Results and Discussion

We evaluated 102 isolates, including 59 *Enterobacterales* carrying *bla*_KPC_ gene (58 *bla*_KPC_ and 1 *bla*_KPC_/*bla*_NDM-1_), identified as *K. pneumoniae* (n = 43), *Serratia marcescens* (n = 11), *Escherichia coli* (n = 3), and *Enterobacter cloacae* complex (n = 2) (Table 1). Besides, it was evaluated as 43 *bla*_KPC_ negative *Enterobacterales*, with 24 of them carrying other carbapenemase genes: 19 *bla*_NDM-1_, 4 *bla*_OXA-48-like_, and 1 *bla*_NDM-1_/*bla*_OXA-48-like_, mainly *K. pneumoniae* (n = 13), but also *E. cloacae* complex (n = 9), *E. coli* (n = 1), and *Klebsiella oxytoca* (n = 1), as shown in Table 2. The *bla*_KPC_-positive isolates had MICs of meropenem ranging from 16 to >256 µg/mL, while the isolates producing carbapenemase other than KPC presented MICs ranging from ≤0.5 to 256 µg/mL.

Moreover, nineteen isolates that had negative results for carbapenemase genes (i.e., KPC, NDM-1, GES, OXA-48, IMP, and VIM) by the HRM-qPCR were also included. They were identified as *K. pneumoniae* (n = 11), *E. cloacae* complex (n = 3), *K. oxytoca* (n = 3), and *Klebsiella. aerogenes* (n = 2) (Table 2), with MICs varying from ≤0.5 to 64 µg/mL.

Statistical results showed a non-normal distribution by Shapiro-Wilk normality test (*p*-value < 0.001). Then, the Mann-Whitney U test confirmed a statistically significant difference (*p*-value < 0.001), considering the presence of KPC peaks when both groups of bacteria (*bla*_KPC_-positive and negative) were compared. The area under the curve (AUC) of the receiver operating characteristic (ROC) curve for this peak was 0.997, which enabled us to believe the detection of KPC peak is capable of significantly discriminating populations of KPC-producing bacteria.

**Figure 1:**
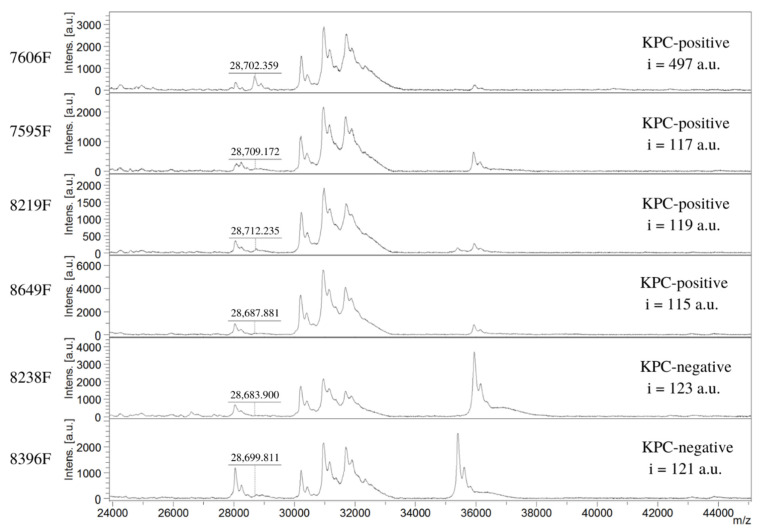
False negative (7595F, 8219F, and 8649F) and false positive (8238F and 8396F) results, considering the visual analysis of the spectra (positive control: 7606F) and the intensities of the peak with the expected *m*/*z* for KPC, compared with HRM-qPCR. I: intensity; a.u.: arbitrary units; MIC: meropenem minimal inhibitory concentration.

We first analyzed results by visual observation after spectra were baseline-subtracted and smoothed. Considering this, 56 out of 59 *bla*_KPC_-positive isolates presented a positive peak in at least one of the triplicates, providing a 94.9% sensitivity. KPC peaks were found at 28,661–28,736 *m*/*z*, with an arithmetic mean ± SD of 28,698 ± 23 (95% CI). If two or three replicates presented a spectrum, the *m*/*z* average of the peak was considered. For three isolates (7595F, 8219F, and 8649F), a clear KPC peak was not found (Figure 1 and Table 1). Of note, all *bla*_KPC_-positive isolates presented meropenem MIC ≥ 16 µg/mL. On the other hand, for the 43 isolates negative to *bla*_KPC_ by HRM-qPCR, the peak was absent (100% specificity) (Table 2).

Previous studies showed different *m*/*z* values for KPC peaks. Moreira et al. presented peaks at *m*/*z* 28,685–28,691 [39]. As determined by Yoon et al., the molecular mass of the intact KPC-2 polypeptide estimated for *bla*_KPC-2_ transformants was 28,718 Da; however, they observed KPC peaks at *m*/*z* 28,708–28,728 corresponding to *m*/*z* of different KPC variants [38]. Indeed, the mass of KPC peak varies according to the enzyme subtype, which may justify the *m*/*z* range observed in our experiments as well as in other studies [38]. In this context, one limitation of our study is that we did not discriminate the KPC subtypes, as the HRM-qPCR detects all recognized KPC genes [40].

**Figure 2:**
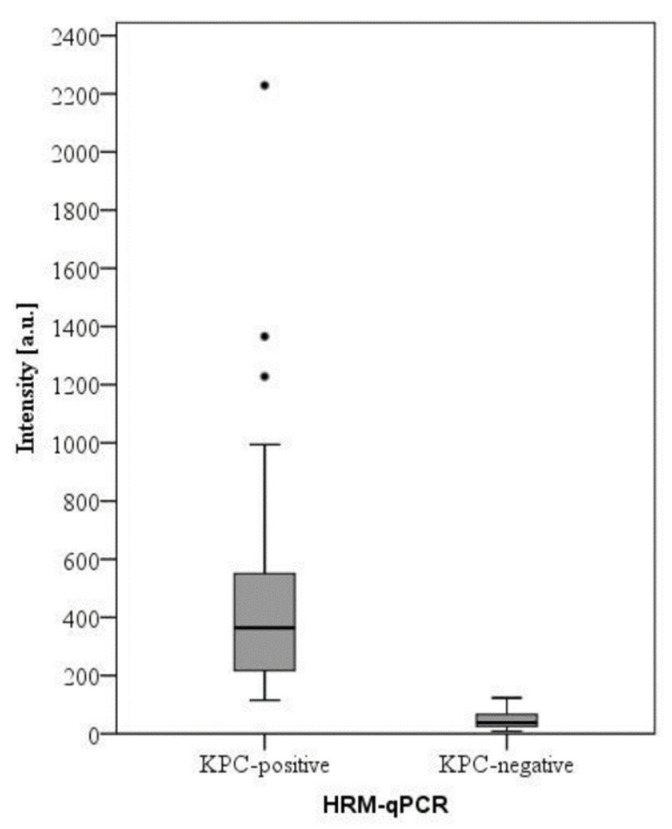
Box plot showing the median (line in the middle) and interquartile range calculated from the intensities of the 28,698 Da peak in the *bla*_KPC_-positive and negative isolates. Dots above the whiskers indicate the outliers. The *x* and *y* axes show the HRM-qPCR result and intensity (arbitrary units—a.u.), respectively.

The second variable we evaluated was intensities of KPC peaks. For *bla*_KPC_-positive isolates, it ranged from 115 to 2229 (a.u.), with a median value of 364 (Figure 2 and Table 1). On the other hand, *bla*_KPC_-negative isolates, despite not having any peaks by visual observation, presented a spectrum with an uneven background at 28,661–28,736 *m*/*z* and very low intensity (9–123; a median value of 38 [a.u.]) (Figure 2 and Table 2).

Considering, as suggested elsewhere [39], that intensity values of 120 (a.u.) or higher indicate a KPC-positive isolate, the method presented a sensitivity of 94.9% and a specificity of 95.3%. Indeed, using this cut-off, 3 false-negative results were observed, which were the same isolates classified as false negative when analysis was performed by visual observation: *K. pneumoniae* isolates 7595F, 8219F, and 8649F (Figure 1 and Table 1), with intensities of 117, 119, and 115 (a.u), respectively. Of note, these values of intensity are borderline considering 120 (a.u.) as the cutoff.

The phenotypic combined disc test confirmed these false-negative isolates as serine-β-lactamases producers, and MIC values were 128 µg/mL or higher. Besides, KPC gene sequencing revealed a closer match with approximately 100% identity in an overlap of 798 nucleotides with the registration under the name “beta-lactamase KPC-2 [*Klebsiella pneumoniae*]”. Thus, the reasons for the intensities just below 120 (a.u.) remain to be elucidated.

On the other hand, among the 43 *bla*_KPC_-negative *Enterobacterales*, we classified 2 isolates as false positives (Figure 1 and Table 2) if the cut-off of 120 (a.u.) was applied: a *K. pneumoniae* (8238F) not carrying carbapenemase genes, with MIC of 1 μg/mL, and a *bla*_NDM-1_-positive *E. cloacae* complex (8396F), presenting MIC of 4 μg/mL. These isolates had intensities of 123 and 121 (a.u.), respectively, even after repetition was performed.

As described above, the intensity values of both false negatives and false positives were very close to the intensity cut-off of 120 (a.u.). For this reason, based on our experience and considering what has been published for carbapenem hydrolysis assays (i.e., intermediate values of logRQ) [27,41], we propose an intermediate value of intensity (i.e., a “buffer” zone), between 115 and 125 (a.u.), in which results would be considered indeterminate or inconclusive. Therefore, applying this “buffer” zone, 7 isolates were classified as indeterminate or inconclusive. These isolates included those 5 bacteria with false-positive and negative results considering the cut-off of 120 (a.u.), but also another 2 isolates, both *S. marcescens* (8848F and 9221F), with intensities of 125 and 121 (a.u.), respectively. Excluding these inconclusive results, the detection of KPC isolates, considering the intensity of KPC peak, would present 100% sensitivity and specificity. It must be highlighted that other studies did not have proposed intermediate values to evaluate the intensity of the KPC peak [37,38].

We are aware that the visual determination of the presence or absence of the KPC peak may be operator-dependent. That is the reason why we suggested the use of the cut-off value of peak intensity. We believe that it is necessary to better differentiate KPC positive and negative isolates. Indeed, establishing a non-ambiguous way to evaluate the MALDI-TOF results for KPC detection, such as a cut-off value of peak intensity, is important to avoid misleading results from the technique. It should be noted, however, that our results do not demonstrate significant issues using the visual analysis of the spectra. Even so, it must be considered that the cut-off value should be standardized for each laboratory to overcome inter-laboratory variations in spectra.

**Figure 3:**
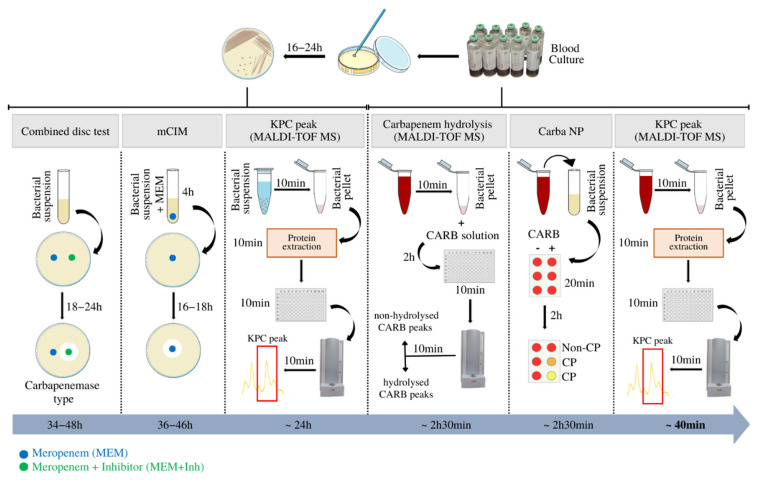
Turnaround time of available alternative methods to detect carbapenemase production among *Enterobacterales*. CARB: carbapenem; CP: carbapenemase producer without definition of type; Non-CP: non-carbenemase producer. The figure was partly generated using Servier Medical Art, provided by Servier, licensed under a Creative Commons Attribution 3.0 unported license.

The rapid identification of carbapenem-resistant organisms is critical not only for the initiation of the proper antimicrobial regimen but also for stopping their spread. The preliminary screening for carbapenemase producers in clinical specimens is traditionally based on phenotypic tests, whereas confirmation tests are mainly based on molecular assays [13]. Conventional phenotypic methods have some important disadvantages, mainly the fact that they are time-consuming, as they require bacterial growth in solid culture medium (18–24 h) plus overnight incubation for test reading (Figure 3). Furthermore, they can be difficult to interpret, and sensitivity/specificity vary between different species [13,16,24,42].

In times where speed in releasing results is increasingly demanded from clinical microbiology laboratories, a few methods, such as the detection of carbapenem hydrolysis by MALDI-TOF MS and Carba-NP (Figure 3), can be performed directly from positive blood cultures, improving the time of carbapenem resistance detection. It may enable the establishment of proper treatment in approximately two and a half hours [14,27,42]. The hydrolysis assay and Carba-NP are not able to determine the carbapenemase type, which may be especially needed when using the new combinations of beta-lactam and beta-lactamase inhibitors [11]. Despite having excellent sensitivity and specificity, immunochromatographic tests are not included in Figure 3 due to the high cost per sample of about 15€, which is considered expensive for some laboratories since the MALDI-TOF detection method can be performed for less than 1€ per sample [17]. Therefore, the strength of our study was to allow the detection of KPC (the most widely disseminated carbapenemase type) from positive blood cultures in less than 1 h from positivity of blood cultures, which would be valuable to improve patient outcome and antimicrobial stewardship.

## 3. Materials and Methods

### 3.1. Bacterial Isolates and Carbapenemase Characterization

A total of 102 clinical *Enterobacterales*, recovered between 2018 and 2021 from patients attending a tertiary hospital in Porto Alegre, southern Brazil, were evaluated. The study was approved by the local research ethical committee (CAAE 167 31638920800005327). Bacteria were identified by MALDI-TOF MS (Bruker Daltonics, Billerica, MA, USA).

Carbapenemase genes were detected by multiplex high-resolution melting real-time PCR (HRM-qPCR) [40]. Briefly, bacterial DNA was extracted by thermal lysis. To do so, the bacteria (from the solid culture medium) were placed in a sterile Eppendorf tube with 500 µL of TE buffer, which was maintained at 80 °C in a dry bath and at −20 °C until freezing. The material was centrifuged, and the supernatant was quantified by Nanodrop Nucleic Acid Quantification (Thermo Fisher Scientific, Waltham, MA, USA) prior to HRM qPCR (MeltDoctor™ HRM Master Mix, Thermo Fisher Scientific). The qPCR multiplex used specific primers, as published elsewhere [40], for the following carbapenemase genes: *bla*_KPC_, *bla*_NDM_, *bla*_OXA-48-like_, *bla*_IMP_, *bla*_GES_, and *bla*_VIM_.

Susceptibility to meropenem (Sigma-Aldrich, St. Louis, MO, USA) was determined by broth microdilution (0.5 to 256 µg/mL), according to ISO 20776-1 [43] and interpreted using the European Committee on Antimicrobial Susceptibility Testing (EUCAST) breakpoints [44]: isolates presenting MIC > 8 μg/mL were considered resistant to meropenem. *Escherichia coli* ATCC 25922 was included for quality control.

Isolates for which the *bla*_KPC_ gene was detected but peak/intensity was not identified were evaluated phenotypically by the enzymatic inhibition test according to the EUCAST guideline [45]. Briefly, a 0.5-McFarland suspension was prepared for each isolate and inoculated on Mueller-Hinton Agar (Sigma-Aldrich). Meropenem discs (OXOID, Hampshire, UK), 10 µg, were used as substrate; in two of them, 10 µL of ethylenediaminetetraacetic acid (EDTA) (NEON, São Paulo, Brazil) 0.1 M or phenylboronic acid (PBA) (Sigma-Aldrich) 40 mg/mL solution was added. After 18 to 24 h of incubation at 35–37 °C, an increase in the inhibition zone of at least 5 mm comparing discs with and without PBA defined the isolate as a KPC producer.

False-negative isolates classified as *bla*_KPC_-positive by HRM-qPCR had their amplicons submitted to Sanger sequencing to confirm the presence of *bla*_KPC_. The results obtained by sequencing were compared with the GenBank database using the “National Center for Biotechnology Information Computer Blast” program (https://blast.ncbi.nlm.nih.gov/Blast.cgi, accessed on 13 December 2022).

### 3.2. Blood Culture Samples

Bacteria were cultured on Mueller-Hinton agar and incubated overnight. To simulate positive blood cultures, 1 mL of a bacterial suspension containing approximately 10^7^ colony forming units (CFU) was inoculated into an aerobic blood culture bottle (BacT/ALERT^®^ FA Plus REF 410851, bioMérieux, Marcy-l’Étoile, France), which had previously been inoculated with 4 mL of human blood [37]. Thereby, a final bacterial concentration of approximately 2 × 10^6^ UFC/mL per bottle was achieved. The bottle was incubated following the manufacturer’s instructions (BacT/ALERT^®^, bioMérieux) until positivity was achieved.

### 3.3. Protein Extraction

Once positive, a bacterial pellet was obtained by successive centrifugation steps [37]. Briefly, 1.4 mL of bottle solution was transferred to an eppendorf tube, which was centrifuged at 1450 rpm (200× *g*) for 5 min. The supernatant was collected and centrifuged at 12,210 rpm (14,170× *g*) for 1 min, and the pellet was washed with distilled water (900 µL), vortexed for 30–60 s, and centrifuged at 13,000 rpm (16,060× *g*) for 2 min. The bacterial pellet was suspended in 300 μL of distilled water and vortexed for 30 s at room temperature. Then, 900 μL of absolute ethanol (Dinamica, São Paulo, Brazil) was added, vortexed for 30–60 s, and centrifuged at 13,000 rpm (16,060× *g*) for 2 min. The supernatant was discarded, and the pellet was re-suspended in 100 µL of extraction solvent (formic acid—isopropyl alcohol—water, 17:33:50 *v*/*v*) (formic acid and isopropyl alcohol from Sigma-Aldrich, Missouri, EUA). The suspension was vortexed for 30–60 s and centrifuged for 2 min at 13,000 rpm (16,060× *g*). The clean supernatant extract was kept at room temperature for further analysis [39].

### 3.4. Target Spot Loading

The extracted protein was spotted onto a steel target plate using a double-layer sinapinic acid (SA) method [37]. The first layer was composed of 0.7 μL of SA-saturated solution (10 mg/mL SA in absolute ethanol) (sinapinic acid from Sigma-Aldrich, Missouri, EUA). For the second layer, protein extract was mixed 1:1 with 10 mg/mL of SA solution in acetonitrile (30:70 *v*/*v*) (Merck, Darmstadt, Germany) and 0.1% trifluoroacetic acid (Sigma-Aldrich) in water. One microliter of this sample/matrix mixture was deposited onto a spot containing the first layer. The sample was left to dry at room temperature and then analyzed by MALDI-TOF MS. Each sample was analyzed in triplicate, loaded once into three different spots (i.e., three spectra per sample).

### 3.5. Spectra Acquisition

Linear MALDI-TOF spectra were obtained in the positive ion mode of the Microflex LT mass spectrometer (Bruker Daltonics, Bremen, Germany) with flexControl 3.4 software (Bruker Daltonics). The parameters were configured as previously described: mass range of 17,000 Da to 50,000 Da; spectrometer ion source 1, 20.08 kV; ion source 2 18.16 kV; lens 6.03 kV; pulsed ion extraction, 550 ns; detection gain of 2803 V; sample rate; and electronic setting of 0.50 GS/s. Laser frequency is 60 Hz, and laser power ranges between 60 and 85%. Each spectrum was obtained after 100 shots per spot. Data were automatically acquired using autoXecute mode, and spectra were analyzed using FlexAnalysis 3.4 software (Bruker Daltonics) [38]. Before each run, the spectrometer was calibrated using Protein Standard II Calibration Mix (Bruker Daltonics).

### 3.6. Data Analysis

We searched for a peak closer to the expected size of the KPC (28,643–28,731 Da), as determined elsewhere [39]. Two parameters were considered. First, the visual presence of the KPC peak was checked. After the relative ion intensity [arbitrary units (a.u.)] was considered after the spectra were baseline subtracted and smoothed, the arithmetic mean ± standard deviation (SD) were calculated, as were sensitivity and specificity, considering the results of HRM-qPCR [40] as a reference. The Shapiro–Wilk test was conducted to test normality, and the Mann–Whitney test was conducted to express differences between the groups. Still, the area under the curve (AUC) of the receiver operating characteristic (ROC) curve was also performed to confirm that the peak found corresponded to *bla*_KPC_-positive isolates. All statistical tests were performed with a 95% confidence interval. Statistical analyses were performed using SPSS (PASW Statistics, version 18.0.3).

## 4. Conclusions

The widespread presence of CRE, especially KPC, has generated an urgent need for the development of rapid and reliable methods for detecting carbapenem resistance. In this context, the potentials of MALDI-TOF MS have been explored. Our study presented an optimized protocol to detect KPC peaks using MALDI-TOF MS directly from positive blood cultures. It proved to be an accurate and rapid method to discriminate between spectra of *Enterobacterales* that produce and do not produce KPC. In order to make the analysis unambiguous, we proposed a cut-off value for the intensity of the KPC peak, as we believe the visual observation of the peak may be subjective in some instances. Applying this methodology, the detection of carbapenem resistance would be very unexpansive and rapid, reducing the turnaround time of the exam for up to 1 day and potentially contributing to the establishment of adequate therapy for patients with infections caused by CRE.

## Figures and Tables

**Table 1:** Characteristics of *bla*_KPC_-positive bacteria used to inoculate blood cultures for KPC detection and results of spectra analysis by direct inspection and determination of intensity.

ID	Species	KPC Peak *m*/*z*	i (a.u.)	Meropenem MIC (µg/mL)
7023F	*E. coli*	28,713	563	64
7431F	*K. pneumoniae*	28,727	351	256
7434F	*K. pneumoniae*	28,663	663	64
7437F	*E. cloacae* complex	28,671	951	64
7440F	*K. pneumoniae*	28,726	1366	256
7442F	*K. pneumoniae*	28,726	994	128
7462F	*K. pneumoniae*	28,717	530	64
7464F	*K. pneumoniae*	28,688	883	>256
7467F	*K. pneumoniae*	28,724	683	128
7480F	*K. pneumoniae*	28,721	434	128
7481F	*K. pneumoniae*	28,729	298	256
7502F	*K. pneumoniae*	28,710	360	>256
7514F	*K. pneumoniae*	28,720	200	128
7590F	*K. pneumoniae*	28,684	584	>256
7592F	*K. pneumoniae*	28,663	2229	128
7593F	*K. pneumoniae*	28,721	149	128
7594F	*K. pneumoniae*	28,665	450	128
7595F	*K. pneumoniae*	Absent	117	>256
7599F	*K. pneumoniae*	28,715	202	128
7605F	*K. pneumoniae*	28,713	224	64
7606F	*K. pneumoniae*	28,679	497	64
8156F	*K. pneumoniae*	28,736	139	16
8219F	*K. pneumoniae*	Absent	119	128
8285F *	*K. pneumoniae*	28,687	364	64
8467F	*K. pneumoniae*	28,721	502	>256
8649F	*K. pneumoniae*	Absent	115	>256
8799F	*S. marcescens*	28,677	1228	>256
8817F	*K. pneumoniae*	28,715	668	64
8829F	*S. marcescens*	28,688	134	256
8848F	*S. marcescens*	28,717	125	>256
8871F	*S. marcescens*	28,674	173	>256
8882F	*K. pneumoniae*	28,731	520	32
8884F	*E. coli*	28,704	264	256
8910F	*S. marcescens*	28,672	245	64
8946F	*S. marcescens*	28,674	210	256
8974F	*E. coli*	28,668	394	256
8982F	*S. marcescens*	28,666	746	128
9077F	*K. pneumoniae*	28,690	237	16
9079F	*S. marcescens*	28,709	131	16
9100F	*K. pneumoniae*	28,677	298	>256
9169F	*E. cloacae* complex	28,715	298	>256
9173F	*K. pneumoniae*	28,722	375	32
9176F	*S. marcescens*	28,699	331	128
9221F	*S. marcescens*	28,677	121	32
9236F	*K. pneumoniae*	28,677	593	128
9274F	*K. pneumoniae*	28,661	414	>256
9279F	*K. pneumoniae*	28,702	310	>256
9283F	*K. pneumoniae*	28,702	796	256
9288F	*K. pneumoniae*	28,661	460	64
9289F	*K. pneumoniae*	28,704	393	128
9294F	*K. pneumoniae*	28,725	855	16
9295F	*K. pneumoniae*	28,670	538	32
9297F	*K. pneumoniae*	28,720	322	256
9299F	*K. pneumoniae*	28,670	173	128
9300F	*S. marcescens*	28,725	270	128
9301F	*K. pneumoniae*	28,664	372	256
9304F	*K. pneumoniae*	28,676	442	256
9305F	*K. pneumoniae*	28,706	336	32
9310F	*K. pneumoniae*	28,707	202	128

i: intensity; a.u.: arbitrary units; * *bla*_KPC_ + *bla*_NDM-1_; absent: intensity lower than 120 (a.u.) and there is no visual presence of the peak.

**Table 2:** Characteristics of *bla*_KPC_-negative bacteria used to inoculate blood cultures for KPC detection and results of spectra analysis by direct inspection and determination of intensity.

ID	Species	HRM-qPCR	KPC Peak *m*/*z*	i [a.u.]	Meropenem MIC (µg/mL)
7282F	*K. pneumoniae*	Negative	Absent	96	4
7452F	*K. pneumoniae*	Negative	Absent	42	64
7523F	*K. pneumoniae*	*bla*_NDM-1_	Absent	68	8
8113F	*K. oxytoca*	Negative	Absent	65	16
8143F	*K. pneumoniae*	Negative	Absent	35	32
8144F	*K. pneumoniae*	Negative	Absent	36	8
8152F	*K. pneumoniae*	Negative	Absent	20	4
8155F	*K. pneumoniae*	Negative	Absent	44	16
8158F	*K. pneumoniae*	Negative	Absent	54	≤0.5
8165F	*K. aerogenes*	Negative	Absent	80	4
8215F	*E. cloacae* complex	Negative	Absent	34	4
8238F	*K. pneumoniae*	Negative	Absent	123	1
8300F	*K. oxytoca*	Negative	Absent	28	4
8311F	*K. pneumoniae*	Negative	Absent	54	≤0.5
8314F	*K. oxytoca*	Negative	Absent	16	4
8333F	*K. pneumoniae*	*bla*_NDM-1_	Absent	112	64
8348F	*K. pneumoniae*	*bla*_OXA-48like_	Absent	31	8
8355F	*E. cloacae* complex	*bla*_NDM-1_	Absent	20	8
8378F	*E. cloacae* complex	*bla*_NDM-1_	Absent	24	8
8382F	*K. pneumoniae*	*bla*_NDM-1_	Absent	9	4
8387F	*E. coli*	*bla*_NDM-1_	Absent	23	≤0.5
8389F	*E. cloacae* complex	*bla*_NDM-1_	Absent	31	16
8396F	*E. cloacae* complex	*bla*_NDM-1_	Absent	121	4
8400F	*K. pneumoniae*	Negative	Absent	95	32
8411F	*K. pneumoniae*	*bla*_NDM-1_	Absent	59	128
8412F	*K. pneumoniae*	*bla*_NDM-1_	Absent	34	64
8414F	*E. cloacae* complex	*bla*_NDM-1_	Absent	28	4
8420F	*E. cloacae* complex	Negative	Absent	101	2
8424F	*E. cloacae* complex	*bla*_NDM-1_	Absent	14	8
8432F	*K. pneumoniae*	*bla*_NDM-1_	Absent	83	8
8435F	*K. aerogenes*	Negative	Absent	38	32
8449F	*K. pneumoniae*	*bla*_NDM-1_	Absent	32	8
8471F	*K. pneumoniae*	*bla*_NDM-1_	Absent	19	1
8474F	*K. pneumoniae*	*bla*_OXA-48like_	Absent	107	16
8478F	*E. cloacae* complex	Negative	Absent	61	8
8481F	*K. pneumoniae*	*bla*_OXA-48like_	Absent	50	16
8485F	*K. oxytoca*	*bla*_NDM-1_	Absent	11	≤0.5
8486F	*E. cloacae* complex	*bla*_NDM-1_	Absent	27	32
8889F	*E. cloacae* complex	*bla*_NDM-1_	Absent	47	64
8890F	*E. cloacae* complex	*bla*_OXA-48like_	Absent	12	8
9110F	*K. pneumoniae*	Negative	Absent	39	64
9112F	*K. pneumoniae*	*bla*_NDM-1_ + *bla*_OXA-48like_	Absent	97	256
9291F	*K. pneumoniae*	*bla*_NDM-1_	Absent	16	2

i: intensity; a.u.: arbitrary units; absent: intensity lower than 120 (a.u.) and there is no visual presence of the peak.

## Data Availability

The data presented in this study are available on request from the corresponding author.
